# A Robust Natural Rubber–Polyzwitterion Composite Hydrogel for Highly Enhanced Marine Anti-Biofouling

**DOI:** 10.3390/gels11030203

**Published:** 2025-03-14

**Authors:** Ye Sun, Dominic John, Yuxin Yan, Xueliang Feng, Qingrong Wei, Chunxin Ma, Zhenzhong Liu, Haimei Mao, Tuck-Whye Wong, Yun Chen

**Affiliations:** 1 State Key Laboratory of Marine Resource Utilization in South China Sea, School of Chemistry and Chemical Engineering, Hainan University, Haikou 570228, China; sunye@hainanu.edu.cn (Y.S.); 24220856020063@hainanu.edu.cn (Y.Y.); 22220856010033@hainanu.edu.cn (X.F.); 2 Taizhou Key Laboratory of Medical Devices and Advanced Materials, Taizhou Institute of Zhejiang University, Taizhou 318000, China; zzliu@zju.edu.cn; 3 Sustainable and Smart Materials Laboratory, Department of Biomedical Engineering and Health Sciences, Universiti Teknologi Malaysia, Johor Bahru 81310, Malaysia; jdominic763@gmail.com (D.J.); wongtuckwhye@utm.my (T.-W.W.); 4 Natural Rubber Research & Development Center of Hainan Province for Deep Processing Products, Ledong 572500, China; qingronghao@126.com; 5 Key Laboratory of Quality Safe Evaluation and Research of Degradable Material, State Administration for Market Regulation, Hainan Academy of Inspection and Testing, Haikou 570203, China; mcmhm@163.com

**Keywords:** marine anti-biofouling, polyzwitterion, natural rubber, hydrogel, high mechanical properties

## Abstract

Polyzwitterion (PZW) hydrogel has excellent marine anti-biofouling performance, but it is difficult to effectively work for a long time in natural seawater due to its weak mechanical strength. In this study, a new natural rubber (NR)-PZW composite hydrogel has been reported for long-term anti-biofouling by simply dispersing NR latex into the poly(sulfobetaine methacrylate) (PSBMA) hydrogel network. First of all, owing to the PZW hydrogel network having an anti-polyelectrolyte effect, this NR-PZW hydrogel can provide outstanding anti-biofouling performance, including broad-spectrum anti-bacteria, anti-algae, and anti-protein properties in marine environments. Furthermore, it has a composited natural rubber nanoparticle with a hydrophilic negatively charged outer protein membrane, which can uniformly disperse in the hydrogel to significantly improve its mechanical properties. Therefore, this composited hydrogel can provide not only highly enhanced tensile strength (0.52 MPa) but also ultra-high breaking elongation (738%), which can effectually resist harsh seawater environments. As a result, the NR-PZW composite hydrogel can achieve excellent anti-biofouling performance for more than 3 months within a real marine environment. This work can provide an excellent, robust polyzwitterionic hydrogel for long-term marine anti-biofouling, which will also inspire new strategies for anti-biofouling materials.

## 1. Introduction

Marine biofouling severely damages underwater facilities and is caused by the attachment and invasion of marine organisms, including bacteria, seaweeds, shellfish, and barnacles [1,2]. Marine biofouling leads to more than USD 15 billion of economic loss per year, especially in shipping, aquaculture, and offshore oil/gas exploitation [3].

To deal with this problem, many anti-biofouling methods have been developed, mainly including physical elimination and chemical bio-killing techniques [4,5]. Physical elimination techniques [4,5,6,7] contain manual or mechanical removal of biological dirt, and recent ultraviolet or ultrasonic-assisted cleaning methods, which are low cost but the process is cumbersome and inefficient. Chemical bio-killing techniques [8,9] can sustain resistance to marine bio-adhesion over a long time via chemical surface coatings by killing the marine creatures, but these methods may damage the marine environment, although the bio-killing regents have evolved from high toxicity (such as Chlorine-based oxidizing biocides, tributyltin, and Zinc sulfide) to low toxicity (such as organic quaternary ammonium salt and inorganic cuprous oxide).

In recent years, different from the traditional methods above, some new eco-friendly biofouling coatings [10] have emerged that can efficiently resist marine biofouling without discharging harmful substances into the marine environment. For example, through designing biomimetic micro-structure surfaces [11,12,13] or low surface energy coatings [14,15,16], marine organisms cannot adhere to the surface easily. Among them, the eco-friendly hydrogel-based anti-biofouling membrane is one of the hotspots in this marine anti-fouling field [17,18]. Hydrogel-based membranes commonly own soft and hydrophilic surfaces, which themselves have a certain degree of anti-bioadhesion and can be easily designed for further improvement of their anti-biofouling performance. Firstly, hydrogels with hydrophilic 3-dimensional networks can contain various nontoxic biomass bio-killing reagents [19,20], which can be control-released for long-term marine anti-biofouling. Furthermore, most recently, new kinds of eco-friendly polyzwitterionic (PZW) hydrogels [21,22,23] have been explored based on their excellent anti-polyelectrolyte effect [24,25,26], which not only can efficiently resist marine biofouling but also do not release harmful materials to damage the marine environment.

However, these PZW hydrogels commonly cannot endure natural marine environments for a long time due to their naturally weak mechanical properties [23,24]. That is, although the PZW hydrogels own outstanding anti-biofouling performance for both micro-organisms and large organisms, their high content of water leads to relatively low mechanical strength, leading to constant invasion of marine organisms and intense external forces from waves/tides in harsh marine conditions. To enhance the mechanical properties of the PZW hydrogel for long-term usage in marine environments, double network (DN) structures [27,28], interpenetrating polymer network (IPN)/semi-IPN structures [29,30,31], and composited with nano-materials [32,33] have been introduced.

**Scheme 1:**
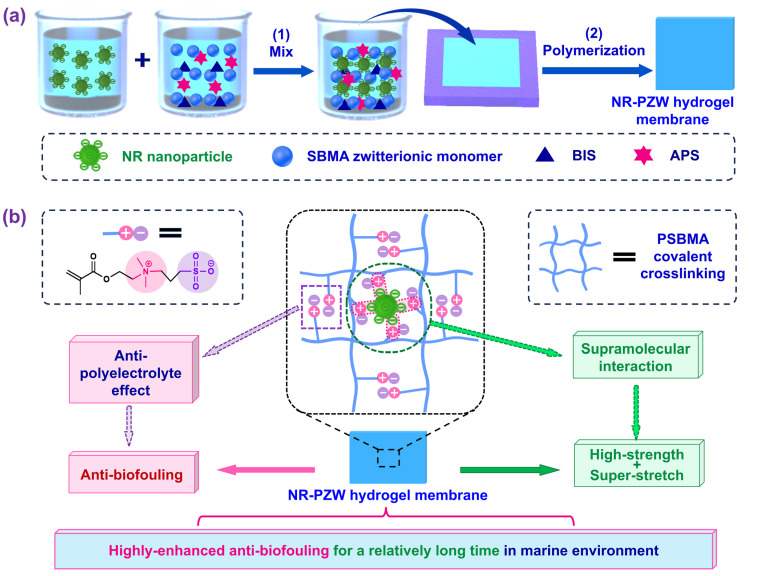
(**a**) The fabrication process of the NR-PZW hydrogel and (**b**) the illustration of the anti-biofouling mechanism and highly enhanced mechanical performance.

Herein, a natural rubber (NR)-PZW composite anti-biofouling hydrogel membrane has been explored by simply mixing the NR latex into the sulfobetaine methacrylate (SBMA) zwitterionic hydrogel precursor solution, which was then free-radical polymerized at 65 °C (Scheme 1). First of all, owing to the PZW hydrogel network with an excellent anti-polyelectrolyte effect [26], this NR-PZW hydrogel can form a dense ultra-hydrophilic water film on the hydrogel surface to efficiently resist bio-adhesion, which can provide excellent broad-spectrum anti-bacteria, anti-algae, and anti-protein properties in marine environments. Furthermore, thanks to the NR nanoparticles of the NR latex with a hydrophilic protein outer shell layer, there can be uniform dispersion in the PZW hydrogel covalent-crosslinked network. The composited NR nanoparticle can form a strong supramolecular interaction between the carboxylate radical groups (negative charges) of its protein shell layer and the quaternary ammonium groups (positive charges) of the PZW polymeric chain, which can provide highly improved mechanical properties including not only highly enhanced tensile strength from 0.04 to 0.52 MPa but also breaking elongation from 94% to 738% [34,35]. Therefore, this as-prepared NR-PZW couples highly efficient marine anti-biofouling and robust mechanical performance for long-term usage. As a result, this NR-PZW composite hydrogel can achieve excellent anti-biofouling performance for more than 3 months within a real marine environment. This work can provide a promising long-term marine anti-biofouling material, which will also inspire new strategies for anti-biofouling materials.

## 2. Results and Discussion

### 2.1. Basic Characterizations of the NR-PZW Hydrogel Membrane

**Figure 1:**
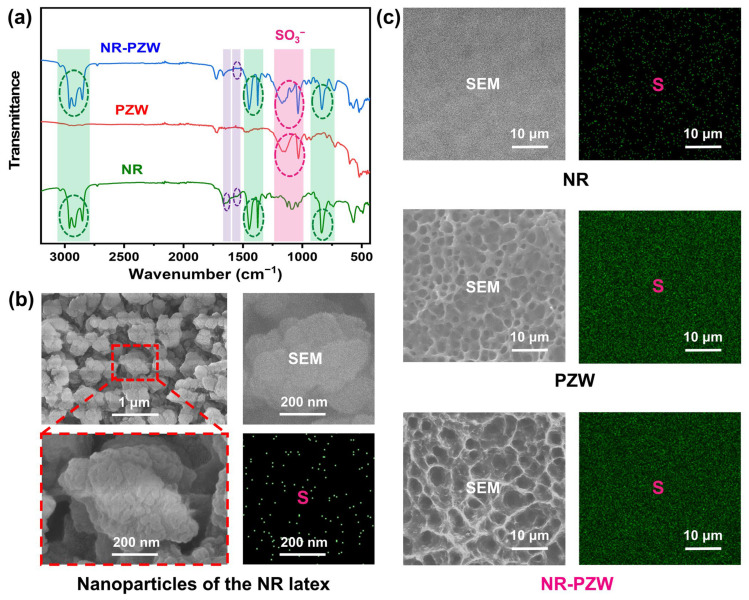
Comparison of (**a**) FT-IR spectra and (**b**,**c**) SEM-connected EDS mappings between the nanoparticle of NR latex, NR sheet, PZW hydrogel, and NR-PZW hydrogel.

The basic chemical component and the morphology of NR-PZW composite hydrogel were researched (Figure 1 and Figure S1–S8). The differences in chemical structure between NR, PZW hydrogel, and NR-PZW hydrogel were evaluated via Fourier transform infrared spectroscopy (FT-IR) (Figure 1a). Firstly, the specific adsorption peaks of NR were tested based on the reported literature [34,35]. (1) Specific peaks of NR’s cis-polyisoprene. Three strong peaks: 2960 cm^−1^ of -CH_3_ asymmetric stretching vibration, 2916 cm^−1^ of -CH_2_ symmetric stretching vibration, and 2851 cm^−1^ of -CH_2_ asymmetric stretching vibration; two strong peaks: 1448 cm^−1^ of antisymmetric deformation vibration peak of methylene, 1375 cm^−1^ of symmetric deformation vibration of methyl; strong single peak: 840 cm^−1^ of C-H out-of-plane deformation vibration on a cis-double-substituted carbon–carbon double bond. (2) Specific peaks of proteins on the surface of the NR nanoparticle. Weak single peak: 1629 cm^−1^ of amide I absorption band and a weak single peak of 1544 cm^−1^ of amide II absorption band. Secondly, the specific adsorption peaks of the PZW were measured: two strong peaks of the sulfonic group including 1033 cm^−1^ and 1064 cm^−1^ in the spectrum of PZW came from the SBMA zwitterionic monomer. Comparison of the characteristic adsorption peaks between the NR, PZW, and NR-PZW samples, the appearance of the specific PZW peaks, and the specific cis-polyisoprene peaks in the spectrum of the NR-PZW indicated that the NR-PZW hydrogel coupled the PZW polymer chain and the NR nanoparticle. Furthermore, the weak single peak (1544 cm^−1^ of amide II specific absorption band) in the spectrum of the NR-PZW further proved the dispersion of NR nanoparticles in the NR-PZW composited hydrogel. In addition, because the content of protein was low in the NR, both of the two specific peaks were weak. Because the content of protein was further reduced in the NR-PZW hydrogel, only the weak single peak (1544 cm^−1^ of amide II specific absorption band) can be found in the spectrum of the NR-PZW. The nanoparticle of the NR latex was observed by scanning electron microscopy (SEM) and the SEM-connected energy-dispersive spectrometer (EDS) mapping (Figure 1b and Figures S1 and S2): the NR nanoparticles were about 500 ± 300 nm of average diameter, and each of them had a wrinkled surface with a high surface area; the SEM-connected EDS mappings showed a clear but slight signal of S (Figure 1b) and N (Figure S2), which confirmed the thin protein of the NR nanoparticle outer layer had a small amount of S (because the inner of NR nanoparticle was made of polyisoprene without S). Furthermore, the NR-PZW was compared with the NR sheet and the PZW hydrogel based on the SEM-connected EDS mappings (Figure 1c and Figures S3–S8). Compared to the slight S element signal intensity of the NR sheet and the ultrahigh S element signal intensity of the PZW hydrogel, the moderate intensity of the S signal of the NR-PZW hydrogel further confirmed that it was composited with NR and PZW. In addition, the cross-section SEM images of the NR-PZW (Figure S7) showed a uniform microporous structure, and the related EDS mappings (Figure S8) also manifested evenly the S signal, which further confirmed the NR nanoparticles were uniformly distributed in the entire volume of NR-PZW hydrogel. That is, NR and PZW can be evenly composited to form the NR-PZW hydrogel, which may explain why although the hydrogelation time was 3 h, the precursor solution state can transform the bulk hydrogel state with the continuous 3D cross-linked PZW network in no more than 10 min (although the reaction is still going on) and the NR nanoparticles (with lower density than water) can be evenly fixed in the cross-linked hydrogel network before the NR nanoparticles aggregating and floating upward.

**Figure 2:**
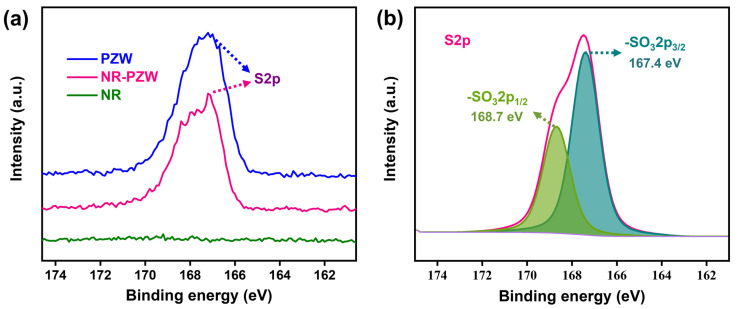
(**a**) The XPS spectra of the PZW, NR-PZW, and NR; (**b**) the XPS peak fitting curve of NR-PZW.

In order to further explore the composition of NR-PZW hydrogels, NR, PZW hydrogels, and NR-PZW hydrogels were compared by X-ray photoelectron spectroscopy (XPS) (Figure 2 and Figure S9). In a comparison of the specific peak of the sulfonic group between the NR sheet, PZW hydrogel, and the NR-PZW hydrogel, there was no specific peak of the sulfonic group in the NR sheet spectrum, while both the PZW hydrogel and the NR-PZW hydrogel had a strong characteristic peak of S2p at (165–170 eV), while there was no signal at the corresponding position of the NR hydrogel spectrum (Figure 2a and Figure S9). This test result indicated that the NR-PZW hydrogel has the PZW hydrogel polymer chain, which had an S element. Furthermore, as shown in the more clear XPS peak fitting curve of NR-PZW hydrogel (Figure 2b), the S2p element signal came from the superposition of two sulfonic acid group signals including S2p_1/2_ at 168.7 eV and S2p_3/2_ at 167.4 eV, which further verified that the S element of NR-PZW hydrogel came from the sulfonic acid group of the PZW polymer chain.

### 2.2. Mechanical Properties of NR-PZW Hydrogel

**Figure 3:**
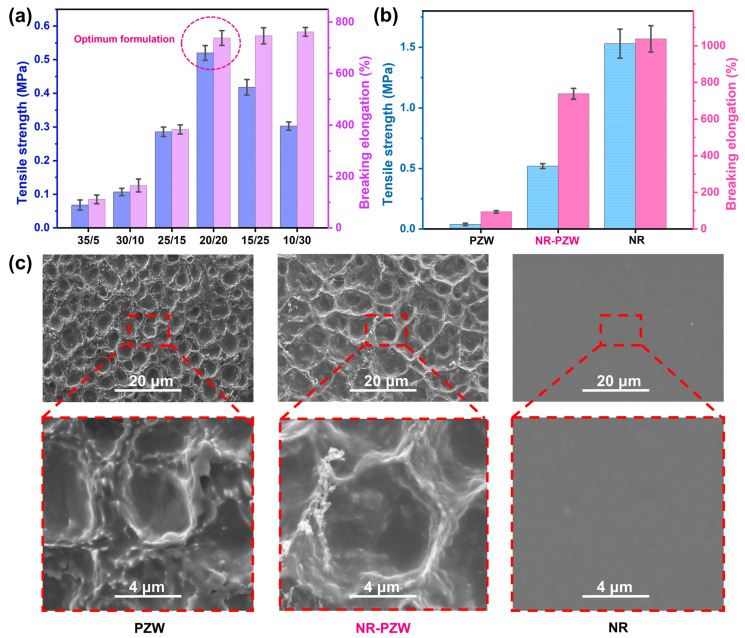
Comparison of mechanical properties between (**a**) NR-PZWs with different formulations and (**b**) PZW, NR-PZW, NR. (**c**) Comparison of SEM images between PZW, NR-PZW, and NR.

The mechanical performance of marine antifouling materials that can withstand wave/tidal erosion and marine biological invasion is an important indicator that must be considered in order to be effectively used in real marine environments for a long time. In particular, hydrogel materials naturally have weak mechanical properties, and it is difficult to maintain long-term integrity and highly efficient anti-bioadhesion in a real seawater environment. In this work, the mechanical properties of the NR-PZW hydrogel were evaluated (Figure 3 and Figures S10–S15, Table S1). A series of NR-PZW hydrogels with a constant 40 wt% of dry mass percentage and different mass ratios of PZW/NR have been systematically researched (Figure 3a and Figure S10) to explore the optimum formulation of the NR-PZW hydrogel for improvement of mechanical properties. With the increase in NR, from m_(PZW)_/m_(NR)_ = 35/5 to 20/20, the mechanical properties (both the tensile strength and the breaking elongation) of NR-PZW hydrogel increased gradually and reached the peak value at m_(PZW)_/m_(NR)_ = 20/20, from 0.07 MPa of tensile strength and 111% of breaking elongation to 0.52 MPa of tensile strength and 738% of breaking elongation. However, with a further increase of NR, from m_(PZW)_/m_(NR)_ = 20/20 to 10/30, the tensile strength decreases gradually, and the breaking elongation remains almost unchanged. In addition, with the change in m_(PZW)_/m_(NR)_, the SEM images of different NR-PZW hydrogels were significantly different, which indicated the large difference in microstructures between different NR-PZW hydrogels (Figure S11). Therefore, the mass ratio of m_(PZW)_/m_(NR)_ = 20/20 can be selected to fabricate the optimum NR-PZW hydrogel for all experiments below without special instruction. Furthermore, the Zeta potential and electrophoretic mobility of the 20 wt% natural rubber aqueous dispersion showed −3.4 ± 0.27 eV and −44.3 ± 0.79 μm cm/V s, which manifested that the NR nanoparticles have relatively strong negative charges based on the carboxylate radical groups on their surface (Figure S12). Therefore, this experiment result may explain that with the increase in NR, the anion–cation interaction between the carboxylate radical groups of the NR nanoparticle protein outer layer and the quaternary ammonium groups of the PZW hydrogel network, can be gradually enhanced to form the added supramolecular interaction of the original PZW hydrogel covalent-crosslinked network, which can significantly enhance the tensile strength; but the PZW largely decreased with a further increase in the NR, which can largely decrease the strength of the PZW hydrogel network and subsequent reduction tensile strength of the NR-PZW hydrogel. In addition, with the increase in NR, this strong supramolecular interaction can firmly and evenly fix the NR nanoparticles with excellent elasticity in the PZW network, which can achieve excellent stress dissipation to reduce stress concentration and, consequently, provide higher and higher tensile strengths. Furthermore, comparison of the mechanical properties of pure PZW hydrogel, that of the NR-PZW hydrogel [m_(PZW)_/m_(NR)_ = 20/20], were significantly enhanced from 0.04 MPa of tensile strength and 94% of breaking elongation to 0.52 MPa of tensile strength and 738% of breaking elongation, which may be due to the composited NR with high tensile strength and high elasticity (Figure 3b) and can be further confirmed by the difference of SEM images (Figure 3c) and mechanical properties (Figures S13 and S14) between them. In addition, a piece of NR-PZW hydrogel with a thickness of 1.5 mm and a width of 1.5 cm was used to lift a bottle of 500 g water (Figure S15), which demonstrated excellent mechanical properties and indicated that it can be used as an anti-biofouling material for long-term usage in the harsh real marine environment.

### 2.3. Swelling–Deswelling Performance of NR-PZW Hydrogel

**Figure 4:**
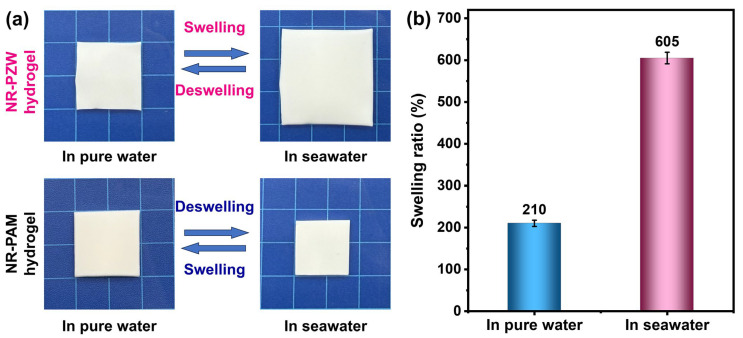
(**a**) Images of NR-PZW hydrogel and NR-PAM hydrogel switching in pure water and seawater; (**b**) swelling ratio of NR-PZW hydrogel within pure water and seawater environment. Each grid size of the photo base plate was 1 cm.

The swelling–deswelling performance of NR-PZW hydrogel was researched in fresh water and seawater (Figure 4 and Figure S16, Table S2). Although common polyelectrolyte-based hydrogels are super-hydrophilic and can provide a dense H_2_O film on their surface for anti-bioadhesion in fresh water, their hydrophilicity can be largely reduced from when they are in pure water to when they are in seawater with high salinity (about 3.5 wt%), and their size will significantly deswell. That is, common polyelectrolyte-based hydrogels cannot achieve a surface-dense H_2_O film in seawater, which cannot provide highly efficient anti-biofouling. This polyzwitterionic NR-PZW hydrogel, different from polyelectrolyte-based hydrogels, can show better hydrophilicity form in fresh water than in seawater, owing to the anti-polyelectrolyte effect, which can provide a denser H_2_O film and better anti-biofouling performance in seawater than in pure water. From pure water to seawater, the size of the NR-PZW hydrogel largely changed by 50% from 2 cm to 3 cm (Figure 4a), and the swelling ratio changed from 210% to 605% (Figure 4b), calculated by Formula (1), which confirmed the NR-PZW hydrogel membrane in seawater can provide better hydrophilicity and a denser water film on its surface than in fresh water and can provide excellent anti-biofouling in theory. That is, this dramatic change can be explained thanks to the poly(sulfobetaine methacrylate) (PSBMA) polymeric network with the synergy of its positive quaternary ammonium groups and negative sulfonic acid groups, which provide excellent anti-polyelectrolyte effects in seawater with high-salinity. In contrast, the NR-polyacrylamide (NR-PAM) composite hydrogel showed significant volume shrinkage from pure water to seawater (its size shrank 20% from 2 cm to 1.6 cm and its swelling ratio decreased from 338% to 214%) (Figure 4b and Figure S16), which may be because it had composited charged NR nanoparticles, similar to the polyelectrolyte. Furthermore, the hydrophilicity of the NR-PZW can be evaluated via comparison of the seawater contact angle change between NR, NR-PZW, and PZW (Figure S17): the NR had relatively poor hydrophilicity with contact angles of 80.3° and 77.6° after 0.5 s and 1.0 s, respectively, while the PZW had excellent hydrophilicity with contact angles of only 24.8° and 21.2° after 0.5 s and 1.0 s, respectively. The NR-PZW had a contact angle of 46.7° and 35.4° after 0.5 s and 1.0 s, respectively, which manifested that the NR-PZW can still maintain excellent hydrophilicity in seawater, although composited with relatively poor hydrophilic NR.

### 2.4. Anti-Biofouling Properties of NR-PZW Hydrogel

**Figure 5:**
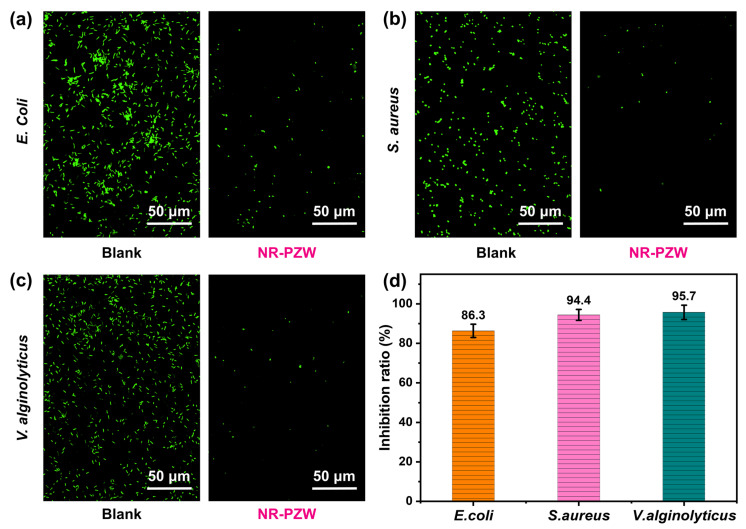
(**a**–**c**) Laser microscope photographs of resistance to different bacterial adhesion of blank samples and NR-PZW hydrogel; (**d**) the inhibition ratio of NR-PZW hydrogel to three kinds of bacteria, respectively.

**Figure 6:**
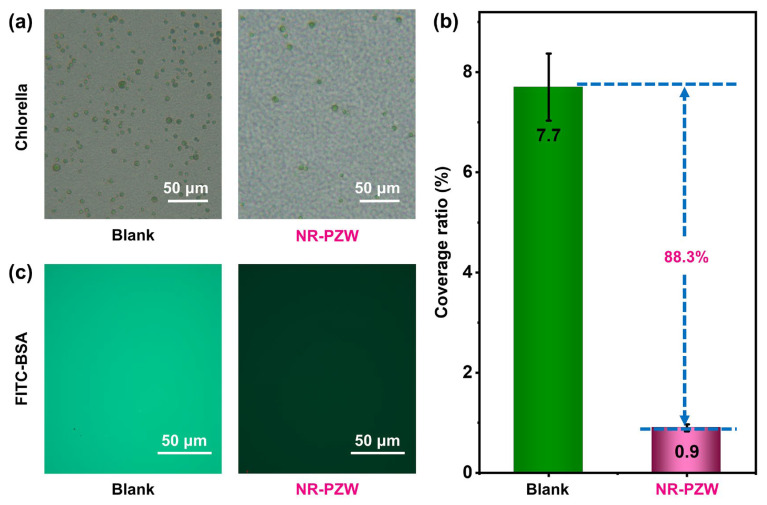
(**a**) Biological microscope photos and (**b**) coverage ratio of chlorella adhesion on the blank hydrogel and the NR-PZW hydrogel; (**c**) fluorescence microscope pictures of FITC-BSA adhesion on blank hydrogel and NR-PZW hydrogel.

Marine biofouling is mainly caused by various marine organisms, such as bacteria, microorganisms, and many other large marine organisms, and the biofouling process is complex and long term [1,2,3]. Generally, when marine equipment or systems without anti-biofouling properties are immersed in the ocean, surface fouling (especially in biofouling) is severe. Firstly, organic matter such as protein, nucleic acid, and polysaccharide will adhere to the surface of the material within a few minutes to form a layer of nutrient base film, and then attract bacteria and microalgae that live on it and form a microbial mucous membrane in about 1 day. After several days of development, it is enough to attract the reproduction of algal spores and the feeding of small organisms. Finally, after several months of continuous biological fouling, large algae and shellfish will take root on the surface of the material and attract large organisms to feed. Once the Marine biological fouling process enters the third step, it will cause irreversible damage to the material, so an important strategy for designing anti-biofouling materials is to delay the formation of the nutrient base membrane and microbial mucosa as much as possible. Hydrogels with high hydrophilicity will form a water film on the surface (the PZW hydrogel can form a denser water film than common hydrogel) when they are in the water environment, which shows unique advantages in this respect. The anti-bioadhesion properties of the NR-PZW hydrogels were researched including anti-bacteria, anti-algae, and anti-protein (Figure 5 and Figure 6) properties, to systematically evaluate the marine anti-biofouling performance of the NR-PZW hydrogel.

The anti-biofouling properties of NR-PZW hydrogels were researched within a simulated marine environment based on real seawater (Figure 5). A series of experiments were designed for the evaluation. Firstly, there were representative Gram-positive bacterium (*Escherichia coli*), representative Gram-negative bacterium (*Staphylococcus aureus*), and unique marine bacterium (*Vibrio alginolyticus*), which were selected to test the anti-bacterial adhesion properties of NR-PZW hydrogel. The preparation procedure of the NR-PAM hydrogel as the control group was the same as that of the NR-PZW hydrogel, except that the zwitterionic monomer SBMA was replaced by acrylamide (AM). In this work, a staining method was selected to test the anti-bacterial adhesion performance of hydrogels: two groups of hydrogel samples were incubated with *E. coli*, *S. aureus,* and *V. alginolyticus*, respectively, and then the surface of the samples was washed with sterilized seawater three times to simulate the scouring effect of ocean waves. Finally, the bacterial adhesion of the samples after staining with green fluorescent nucleic acid stain (SYTO-9) was observed by laser confocal microscopy. In addition, in order to better simulate the marine environment, a real seawater-based culture medium was specially used for bacteria cultivation. As can be seen from the confocal laser photos, the surface of the NR-PAM hydrogel as the control group showed serious *E. coli* adhesion, while only a small number of bacteria appeared on the surface of the NR-PZW hydrogel, and the calculated inhibition ratio of *E. coli* reached 86.3% (Figure 5a,d). Similar effects were seen in confocal laser images of S. aureus and V. alginolyticus, where NR-PZW hydrogel showed even better inhibition ratios of 94.4% and 95.7% against these two bacteria, respectively, indicating that it was a highly effective broad-spectrum anti-bacterial adhesion material in the ocean (Figure 5b–d). The excellent anti-bacterial adhesion performance of NR-PZW hydrogels may be attributed to the super-hydrophilic charged groups of the PZW network, which can produce strong hydration with water molecules to form a dense hydration water surface layer on the NR-PZW hydrogel to resist bacterial adhesion. In addition, NR-PZW hydrogels become more hydrophilic in seawater because of the anti-polyelectrolyte effect, and bacteria need to pass through the dense water layer, which can highly efficiently resist the adhesion of bacteria and most bacteria can be washed away by waves in the real marine environment. On the contrary, the NR-PAM hydrogel without an anti-polyelectrolyte effect cannot form a dense water film in seawater; therefore, it just showed relatively weak anti-bacterial properties (Figure 5).

The anti-algae and anti-protein performance of the NR-PZW hydrogels was also researched within the natural marine environment (Figure 6). Microalgae is one of the main components of microbial mucosa formed in the second stage of marine biological fouling, so the anti-algae adhesion performance of hydrogels is a necessary index to investigate. In this work, chlorella incubated in a seawater medium was selected to test the anti-algal adhesion performance of the NR-PZW hydrogel. The hydrogel samples were taken out after being immersed in chlorella (it was selected as a representative of algae) solution for 3 days. The surface of the samples was washed with seawater 3 times, and then the adhesion of chlorella was observed under a microscope (Figure 6a). Microscopic photos showed that the NR-PAM hydrogel as the control group had serious algal adhesion, and the coverage ratio of chlorella on its surface was 7.7%, calculated by ImageJ (version 1.8.0) software; however, the surface of the NR-PZW hydrogel only had a small number of chlorella, and the corresponding coverage ratio was only 0.9% (Figure 6b). The anti-alga adhesion performance of the NR-PZW hydrogel was 88.3% higher than that of the control group, indicating that the dense hydration layer formed by the hydration of charged groups and water molecules on the PZW network can effectively resist the adhesion of microalgae.

Protein is one of the most important nutrients in the ocean for bacteria and microalgae, which can adhere to the surface of materials in a very short time to form a nutrient base film, which is the basic condition for the occurrence of marine biological fouling. By inhibiting the adhesion of organic matter, the development of subsequent fouling of materials can be effectively slowed down and the service life can be extended. In this work, fluorescein isothiocyanate (FITC)-labeled bovine serum albumin (BSA) was selected as a representative to test the anti-protein adhesion performance of NR-PZW hydrogel. The anti-adhesion of the NR-PZW hydrogel resisting on the BSA was evaluated by fluorescence intensity observed on the surface of the hydrogel under a fluorescence microscope, within a BSA dispersion for 24 h. As shown in Figure 6c, NR-PAM hydrogel as the control group showed bright green strong fluorescence, indicating the presence of a large amount of BSA. In contrast, very weak fluorescence on the surface of NR-PZW hydrogels indicated very little BSA adherence. The NR-PZW hydrogel wrapped in a dense hydration layer can effectively resist protein adhesion, and its excellent marine anti-biofouling performance was proved by combining the previous anti-bacterial adhesion and anti-algal adhesion results.

### 2.5. Anti-Biofouling Properties of NR-PZW Hydrogel in Real Marine Environment

**Figure 7:**
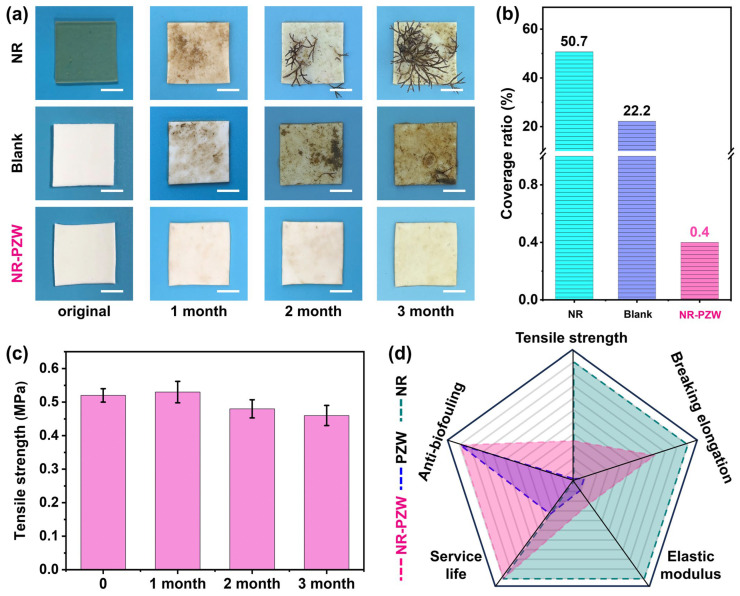
(**a**) Marine experiment photos and (**b**) coverage ratio of smeared area on the NR, blank hydrogel, and the NR-PZW hydrogel; (**c**) variation of tensile strength of NR-PZW hydrogel during marine experiments; (**d**) comprehensive performance comparison of NR, PZW hydrogel, and NR-PZW hydrogel. Scale bar, 3 cm.

Anti-biofouling materials face more complex adhesion types, gnawing by large organisms, and wave impact in the real marine environment, and these effects are difficult to simulate in the laboratory. In order to test the anti-biofouling properties of NR-PZW hydrogel in practical applications, a real marine test was conducted in the sea area near Haikou for 3 months. A total of four groups of samples of PZW hydrogel, NR, NR-PAM hydrogel, and NR-PZW hydrogel were used for real marine experiments. Except for PZW hydrogel, the sampling frequency was once every 10 days, and the sampling frequency of other samples was once every month. As can be seen from Figure 7a, NR was attached with a large amount of dirt and algae thriving on its surface after 3 months of marine testing. The smeared area was calculated by ImageJ software as high as 50.7% (Figure 7b). This was due to the frost-spraying effect of NR after prolonged immersion in seawater, where hydrophilic substances such as proteins migrated to the surface to form a nutrient-rich thin layer, causing NR, which already lacked anti-biofouling properties, to attract microbial aggregation and growth. As a blank sample, NR-PAM hydrogel was also very dirty and visible to the naked eye with algae and large colonies, with a stain area of 22.2%, indicating that the anti-biofouling performance of ordinary hydrogels in the ocean was limited. In contrast, the surface of NR-PZW hydrogel had only a few slight discoloration areas, and the dirt area was only 0.4%, which proved that the dense hydration layer on its surface could effectively resist the adhesion of various organisms and limited the occurrence of subsequent biofouling. In addition, the NR-PZW hydrogel remained intact after three months of sea shock, and its mechanical properties only slightly decreased from 0.52 MPa to 0.46 MPa (Figure 7c). On the contrary, although PZW hydrogel had excellent anti-biofouling properties, its weak mechanical properties made it break in a short time and disappear within 1 month (Figure S18). As shown in the comprehensive performance comparison diagram (Figure 7d), the NR-PZW hydrogel can integrate the highly enhanced mechanical properties of NR and the excellent anti-biofouling performance of the PZW hydrogel together. Therefore, this NR-PZW hydrogel can provide excellent anti-biofouling behavior for relatively long-term usage in real marine environments, which is outstanding among existing hydrogel-based anti-biofouling materials (Table 1) [10,22,23,29,36,37,38,39,40,41].

## 3. Conclusions

In summary, by simply dispersing hydrophilic natural rubber nanoparticles into the PZW hydrogel network, a new NR-PZW hydrogel membrane has been presented for marine anti-biofouling. This hydrogel can be obtained simply by mixing the NR latex into the sulfobetaine methacrylate (SBMA) zwitterionic hydrogel precursor solution before free-radical polymerization. Firstly, owing to the PZW network with an excellent anti-polyelectrolyte effect, this hydrogel can form a dense ultra-hydrophilic water film on the hydrogel surface to efficiently resist bio-adhesion, which is due to excellent broad-spectrum anti-bacteria, anti-algae, and anti-protein properties in the marine environment. Furthermore, the NR nanoparticles of the NR latex with hydrophilic protein outer shell layer can uniformly disperse in the PZW hydrogel covalent-crosslinked network. The composited NR can further form strong supramolecular interaction between the carboxylate radical groups (negative charges) of its protein shell layer and the quaternary ammonium groups (positive charges) of the PZW polymeric chain, which can provide highly enhanced mechanical properties including tensile strength from 0.04 to 0.52 MPa and breaking elongation from 94% to 738%. Therefore, this NR-PZW couples the highly efficient marine anti-biofouling and robust mechanical performance for long-term usage. As a result, this NR-PZW composite hydrogel can achieve excellent anti-biofouling performance for more than 3 months within a real marine environment. This work can provide a promising long-term marine anti-biofouling material, which will also inspire new strategies for anti-biofouling materials.

## 4. Materials and Methods

### 4.1. Materials

Sulfobetaine methacrylate (SBMA, zwitterionic monomer 98.5%, which can form the PSMBA hydrogel network), acrylamide (AM, monomer, 97.5%), N,N′-methylene bisacrylamide (BIS, 98.0%), and ammonium persulfate (APS, 99%) were bought from Aladdin Biochemical Technology Co., Ltd. (Shanghai, China). The natural rubber latex (NR, 60%) was purchased from Huang Chunfa Co., Ltd. (Sadat, Thailand). Each of the agents was utilized directly in the experiments. The seawater was taken from the offshore sea near Ledong County, China.

### 4.2. Characterization

The molecular structure information of the samples was obtained by Fourier transform infrared spectroscopy (FT-IR, Nicolet Summit X, Thermo Scientific, Waltham, MA, USA). The chemical components of the samples were measured using X-ray photoelectron spectroscopy (XPS, 2c50Xi, Thermo Escalab, Waltham, MA, USA). The microstructures and morphologies of samples were observed by scanning electron microscopy (SEM, S-4800, Hitachi, Tokyo, Japan), and elemental quantitative analysis was performed by SEM-connected energy-dispersive spectrometer (EDS) mapping. The mechanical properties of these samples were evaluated via a universal material testing machine (MXL-50, Taso Testing Equipment Co., Ltd., Suzhou, China). The hydrophilicity of these samples was tested using a contact angle measuring instrument (FT-CAMA2, Feitang Testing Equipment Co., Ltd., Suzhou, China). The anti-bacterial adhesion properties of the samples were calculated by a laser confocal microscope (LSM900, Zeiss, Oberkochen, Germany).

### 4.3. Experimental Methods

#### 4.3.1. Preparation of NR-PZW Composite Hydrogel

The NR-PZW composite hydrogel can be prepared simply: Firstly, the concentration of natural rubber latex was diluted from 60% to 25% with pure water, taking 0.4 mL and 100 mg zwitterionic monomer SBMA to fully mix, and then 1 mg cross-linking agent BIS and 1 mg initiator APS were put in successively and mixed to get the SBMA precursor aqueous solution. Second, the SBMA precursor was poured into a reaction tank composed of silicone rubber sheets and glass sheets to be sealed for polymerization at 65 °C for 3 h and then dried in an oven at 65 °C. Finally, the hydrogel was soaked in 500 mL of pure water (changed every 4 h) to remove impurities. After purification, the as-prepared robust NR-PZW hydrogel with evenly dispersed rubber nanoparticles was achieved, which were kept in seawater before being tested. In addition, a series of composite hydrogels with different NR/ZWs were prepared to explore the best mechanical properties. At the same time, in order to explore the marine anti-biofouling properties of the NR-PZW composite hydrogels, the blank control group NR-PAM composite hydrogels with SBMA replaced by AM were prepared.

#### 4.3.2. Swelling Performance Test of NR-PZW Composite Hydrogel

The NR-PZW composite hydrogel and NR-PAM composite hydrogel as the control group were, respectively, put into pure water to reach swelling equilibrium. They were cut into square hydrogel sheets with side length of 2 cm and weighed to obtain wet weight ($W_{w}$) in pure water. Then, they were put into seawater to achieve swelling equilibrium and weighed again to obtain wet weight in seawater. Finally, the dry weight ($W_{d}$) of the hydrogels was obtained by repeatedly soaking in pure water to remove salt and drying and weighing. The swelling ratio (SR) of the hydrogels can be obtained by Formula (1):

(1) $$SR={[W}_{w}/W_{d}]\times 100\%$$

#### 4.3.3. Antibacterial Adhesion Test of NR-PZW Composite Hydrogel

Three representative kinds of bacteria were selected including *Escherichia coli*, *Staphylococcus aureus*, and *Vibrio alginolyticus*, which were used to test the adhesion behaviors on the surface of different samples based on the staining method. The three bacteria were added to the sterilized seawater culture medium and incubated at 200 rpm in a constant temperature shaking bed at 37 °C for 24 h. The disc-like samples with a uniform 1.0 cm of diameter were sterilized under an ultraviolet lamp for 30 min on both sides, then washed with sterile PBS solution and dried and placed in 24-well plates. The bacterial solution was adjusted to 10^7^ CFU/mL with sterile PBS solution whose concentration can be calculated via a turbidimeter, and then each hole of the co-incubation sample was injected with 2.0 mL of this solution. The hydrogel samples were incubated at 200 rpm at 37 °C for 6 h, and the surface was washed with sterilized seawater 3 times. A total of 200 μL of diluted SYTO-9 dyeing solution was dropped onto the sample surface and stained for 15 min at 37 °C. The stained hydrogel sample can be tested via the laser confocal microscope to observe the anti-bacterial adhesion performance. The antibacterial adhesion properties of hydrogels can be obtained by Formula (2):

(2) $$IR={[(N}_{b}-N_{a})/N_{b}]\times 100\%$$

where “*IR*” is the inhibition ratio and “$N_{b}$” and “$N_{a}$” are the quantity of bacteria on the surface of blank control hydrogel and anti-biofouling hydrogel, respectively.

#### 4.3.4. Anti-Algae Adhesion Test of NR-PZW Composite Hydrogel

Chlorella was selected to test the anti-algal adhesion performance of hydrogels. Chlorella was inoculated into BG-11 medium solution prepared with seawater and incubated at 25 °C in alternating light/darkness every 12 h for 7 days. The hydrogel discs with a diameter of 1 cm were immersed in the cultured chlorella solution and cultured for 3 days in a constant temperature incubator at 25 °C in alternating light/dark mode. After that, the samples were taken out and washed with seawater 3 times, and the adhesion of chlorella on the surface was observed under a microscope (M330-HD228S, Shenzhen AOSVI Optical Instrument Co., Ltd., Shenzhen, China). The anti-algae adhesion performance of the hydrogel can be evaluated by calculating the coverage ratio of chlorella on its surface by ImageJ software.

#### 4.3.5. Anti-Protein Adhesion Test of NR-PZW Composite Hydrogel

Bovine serum albumin (BSA) was selected to test the anti-protein adhesion of the hydrogel by fluorescence staining. Firstly, BSA solution containing FITC fluorescent labeling was prepared and diluted to 0.5 mg/mL with sterile PBS solution. Then, a 1.0 cm diameter of disc-like hydrogel sample was incubated within the BSA solution on the shaking table (180 rpm) at 25 °C for 24 h. Consequently, fluorescence signals on the sample surface can be tested through a fluorescence microscope (Olympus IX51, Olympus, Tokyo, Japan) to assess the anti-adhesion performance of the sample against the protein.

#### 4.3.6. Real Marine Anti-Biofouling Test of NR-PZW Composite Hydrogel

Four sets of samples of NR, PSBMA hydrogel, NR-PAM composite hydrogel, and NR-PZW composite hydrogel were prepared for real marine experiments. All the samples were first immersed in real seawater to reach swelling equilibrium, then cut to the same size, and then put into the South China Sea near Haikou City to test the anti-biofouling properties. All samples were photographed every month and one NR-PZW composite hydrogel sample was taken for a mechanical properties test. Considering the fragile mechanical properties of PZW hydrogel, the frequency of observation was increased to once every 10 days. The whole test cycle was 3 months, and the anti-biofouling properties of these samples can be obtained by using ImageJ (version 1.8.0) software to calculate the stained areas on the surface.

## Figures and Tables

**Table 1:** Comparison of properties of NR-PZW hydrogel with existing anti-biofouling hydrogels.

Ref	Hydrogels	Tensile Strength (MPa)	Breaking Elongation (%)	Anti-Bacterial	Anti-Algae	Anti-Protein	Marine Test (d)
This work	NR-PZW	0.52	738	√	√	√	90
[29]	PSBMA/PAA	0.4	251	√		√	
[36]	COHG	0.1	310		√		
[37]	amphiphilic	0.1	23		√	√	30
[38]	MOF/SA	0.4	580	√	√	√	
[22]	PSBMA/PVA	2.4	420	√	√	√	180
[39]	SBMA/PVA/SA	0.1	42	√		√	
[23]	ZW/PAO	0.1	63	√			25
[40]	TA/PVA	0.4	228	√	√		90
[41]	PEG	0.4	1600	√		√	120
[10]	Gel/TA/Si	1.0	197	√	√		180

All these references were placed based on the time order. PAA: poly(acrylic acid); COHG: charged organo-hydrogels; SA: sodium alginate; PVA: poly(vinyl alcohol); PAO: poly(amidoxime); TA: tannin acid; PEG: poly(ethylene glycol).

## Data Availability

The original contributions presented in this study are included in the article/Supplementary Material. Further inquiries can be directed to the corresponding authors.

## Supplementary Materials

The following supporting information can be downloaded at https://www.mdpi.com/article/10.3390/gels11030203/s1, Figure S1. SEM mages of the nanoparticles of the natural rubber latex; Figure S2. SEM-connected EDS-mapping of the nanoparticle in the natural rubber latex; Figure S3. SEM-connected EDS-mapping of the NR sheet; Figure S4. SEM-connected EDS-mapping of the PZW hydrogel; Figure S5. SEM-connected EDS-mapping of the NR-PZW hydrogel; Figure S6. Comparison of S-element signal about EDS mapping between NR, PZW, and NR-PZW; Figure S7. Cross-section SEM images of the NR-PZW hydrogel; Figure S8. Cross-section SEM images of the NR-PZW hydrogel; Figure S9. XPS full spectrum of NR-PZW hydrogel; Figure S10. Comparison of tensile properties between the NR-PZW composite hydrogels with different dry mass ratios of the SBMA: natural rubber; Figure S11. SEM images of PZW, NR, and different NR-PZW; Figure S12. Zeta potential and electrophoretic mobility of the 20 wt% natural rubber aqueous dispersion; Figure S13. Comparison of mechanical properties between the PZW, NR-PZW, and NR; Figure S14. Comparison of Young’s modulus between the PZW, NR-PZW, and NR; Figure S15. Photos of NR-PZW lifting heavy objects; Figure S16. Comparison of swelling ratio of NR-PAM hydrogel between pure water and seawater; Figure S17. Comparison of seawater contact angle change between the NR, NR-PZW, and the PZW samples. Figure S18. Marine experiments photos of PZW (scale bar: 3.0 cm); Table S1. Formulations of different NR-PZW hydrogels; Table S2. Comparison of swelling ratio and water content of NR-PZW hydrogel in pure water and seawater.
