# Supplementary material for: A Robust Natural Rubber–Polyzwitterion Composite Hydrogel for Highly Enhanced Marine Anti-Biofouling
Source: Gels. 2025 Mar 14;11(3):203. doi: 10.3390/gels11030203 (PMC11942406; doi:10.3390/gels11030203)
Supplement: Supplementary file 1 [file gels-11-00203-s001.zip › gels-3487761-supplementary.pdf]

A Robust Natural Rubber–Polyzwitterion Composite Hydrogel for Highly Enhanced Marine Anti-Biofouling

Ye Sun [1,2], Dominic John[3], Yuxin Yan [1], Xueliang Feng[1,4], Qingrong Wei[4], Chunxin Ma[1,2,*], Zhenzhong Liu[2], Haimei Mao[5], Tuck-Whye Wong[3] and Yun Chen[1,*]

[1] State Key Laboratory of Marine Resource Utilization in South China Sea, School of Chemistry and Chemical Engineering, Hainan University, Haikou 570228, China; sunye@hainanu.edu.cn (Y.S.); 24220856020063@hainanu.edu.cn (Y.Y.); 22220856010033@hainanu.edu.cn (X.F.)
[2] Taizhou Key Laboratory of Medical Devices and Advanced Materials, Taizhou Institute of Zhejiang University, Taizhou 318000, China; zzliu@zju.edu.cn
[3] Sustainable and Smart Materials Laboratory, Department of Biomedical Engineering and Health Sciences, Universiti Teknologi Malaysia, Johor Bahru 81310, Malaysia; jdominic763@gmail.com (D.J.); wongtuckwhye@utm.my (T.-W.W.)
[4] Natural Rubber Research & Development Center of Hainan Province for Deep Processing Products, Ledong 572500, China; qingronghao@126.com
[5] Key Laboratory of Quality Safe Evaluation and Research of Degradable Material, State Administration for Market Regulation, Hainan Academy of Inspection and Testing, Haikou 570203, China; mcmhm@163.com
* Correspondence: machunxin@hainanu.edu.cn (C.M.); chenyunhappy@hainanu.edu.cn (Y.C.); Tel.: +86-188-5811-5460 (C.M.); +86-186-8983-6813 (Y.C.)

This PDF file includes the following:

Supplementary Materials and Methods

Figure S1 to S14

Table S1 to S2

Supporting Figures

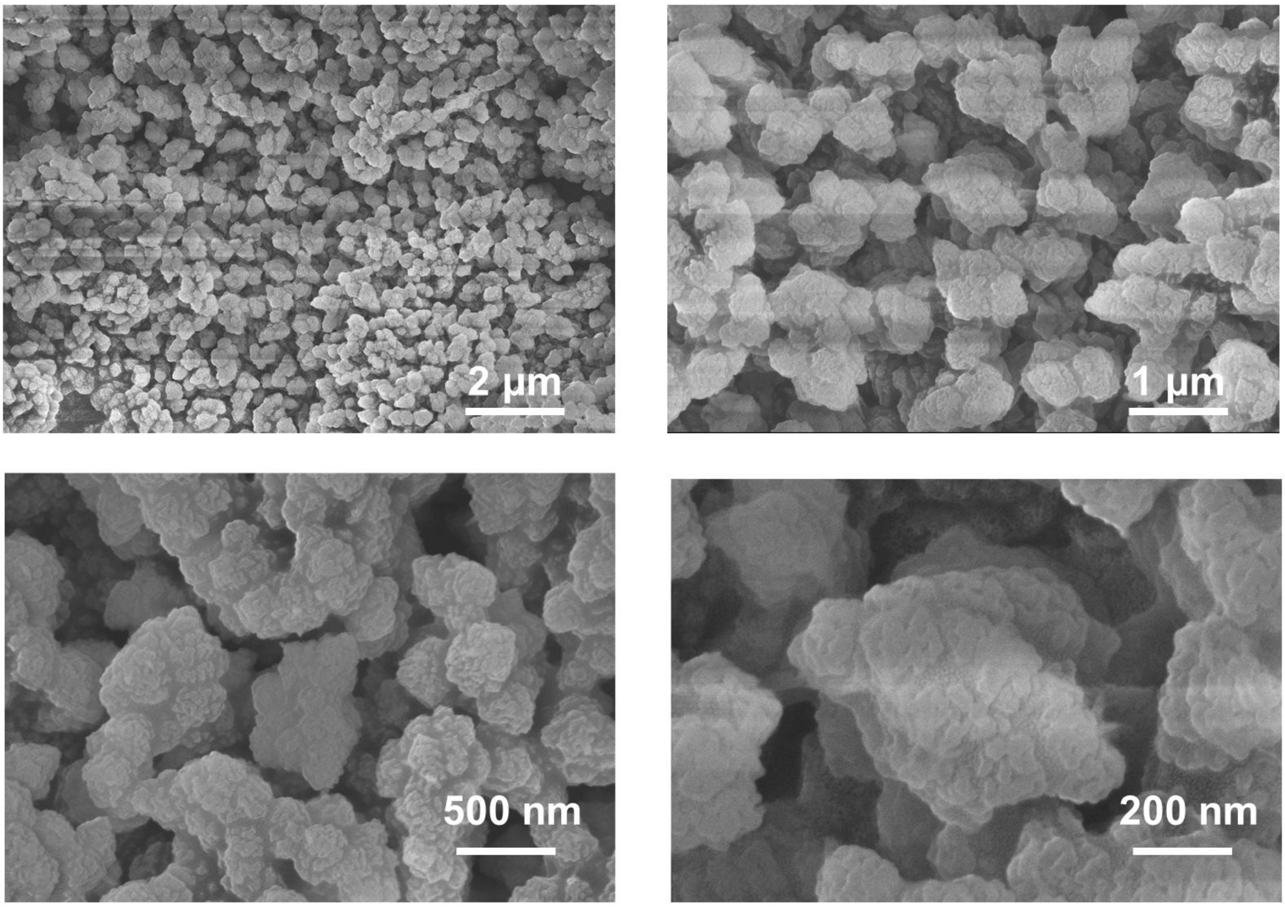

Figure S1. SEM mages of the nanoparticles of the natural rubber latex.

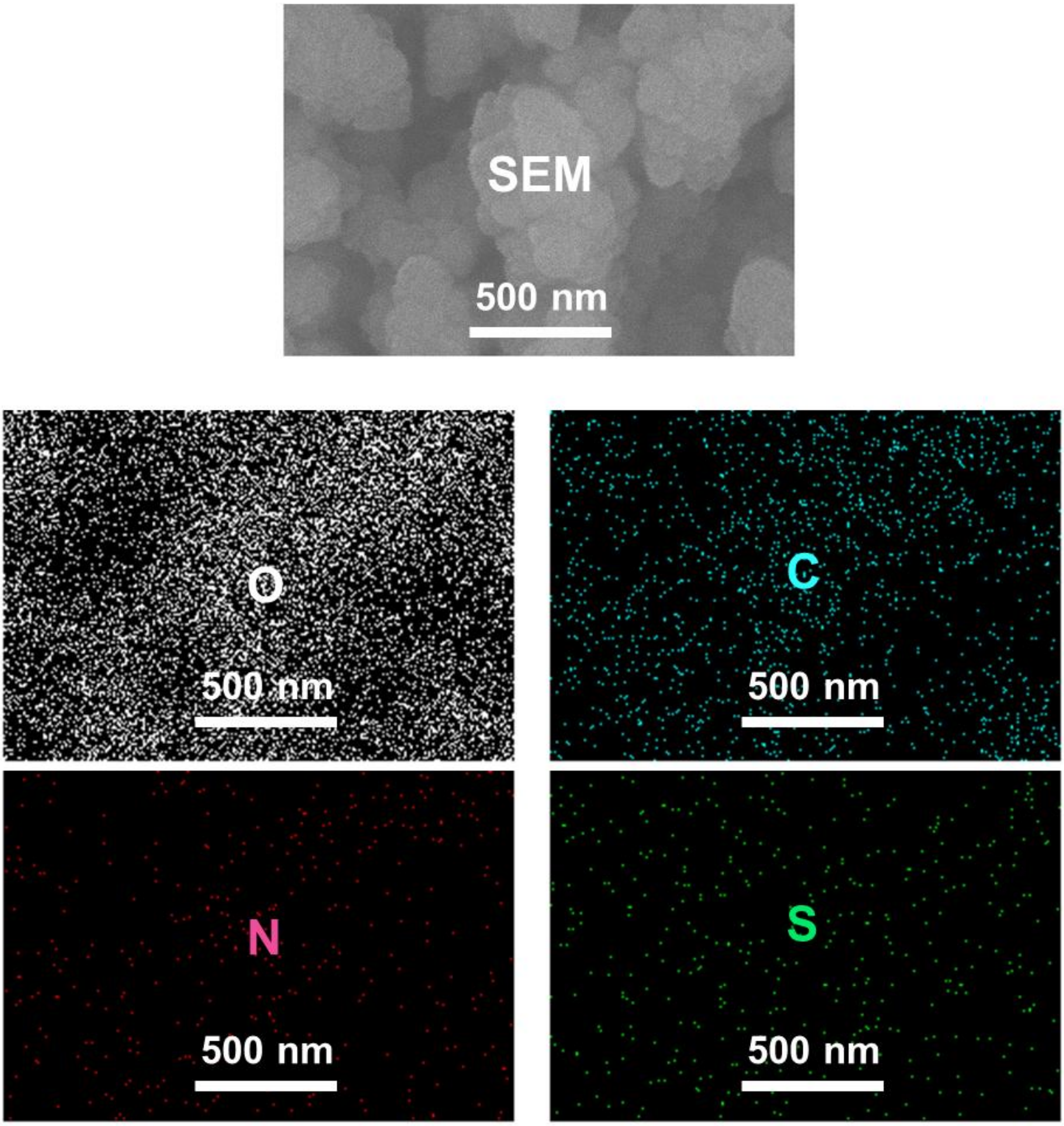

Figure S2. SEM-connected EDS mapping of the nanoparticle in the natural rubber latex.

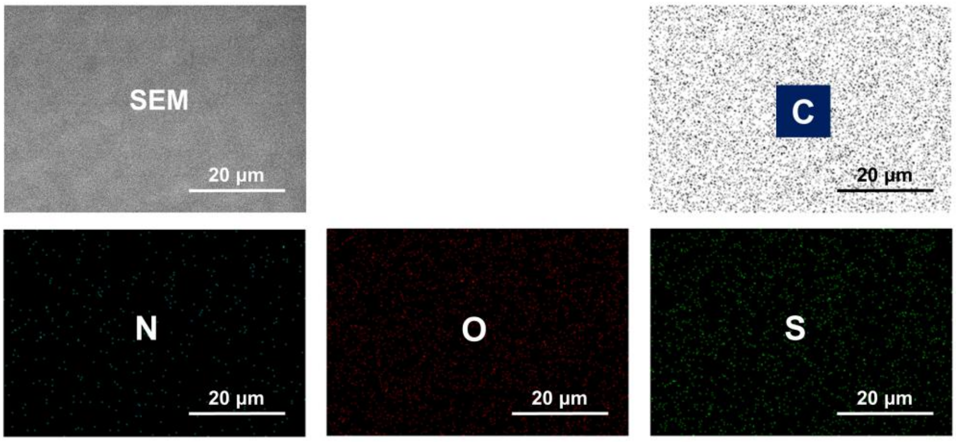

Figure S3. SEM-connected EDS mapping of the NR sheet.

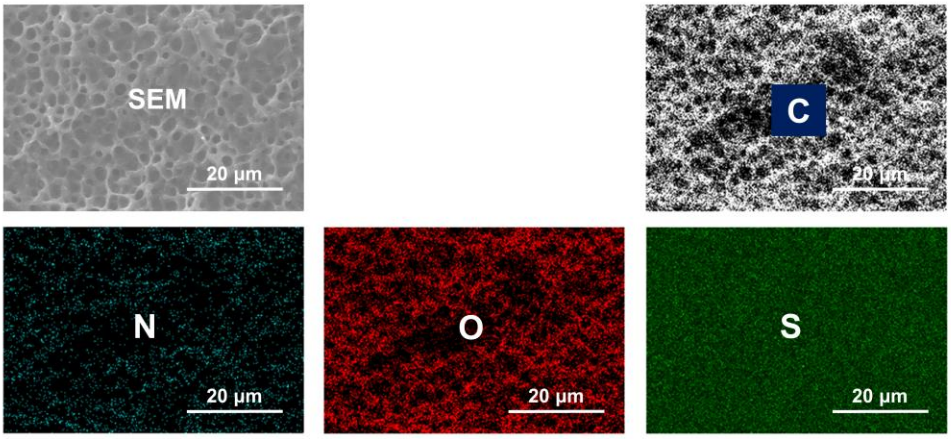

Figure S4. SEM-connected EDS mapping of the PZW hydrogel.

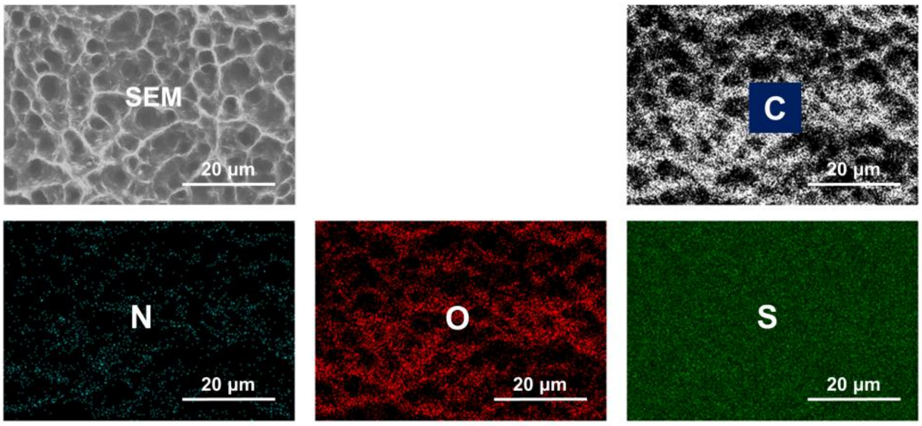

Figure S5. SEM-connected EDS mapping of the NR-PZW hydrogel.

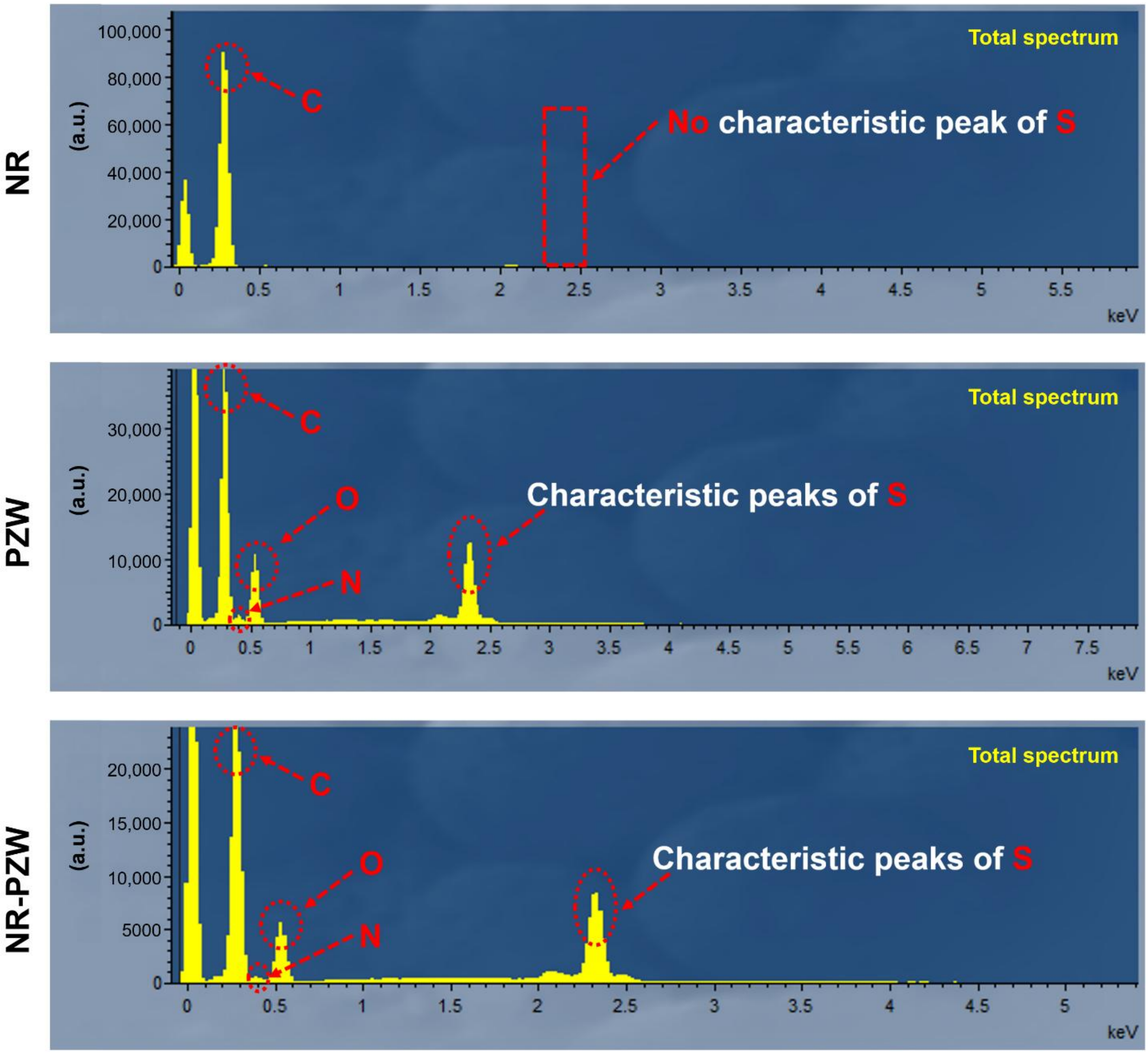

Figure S6. Comparison of S-element signal about EDS mapping between NR, PZW, and NR-PZW.

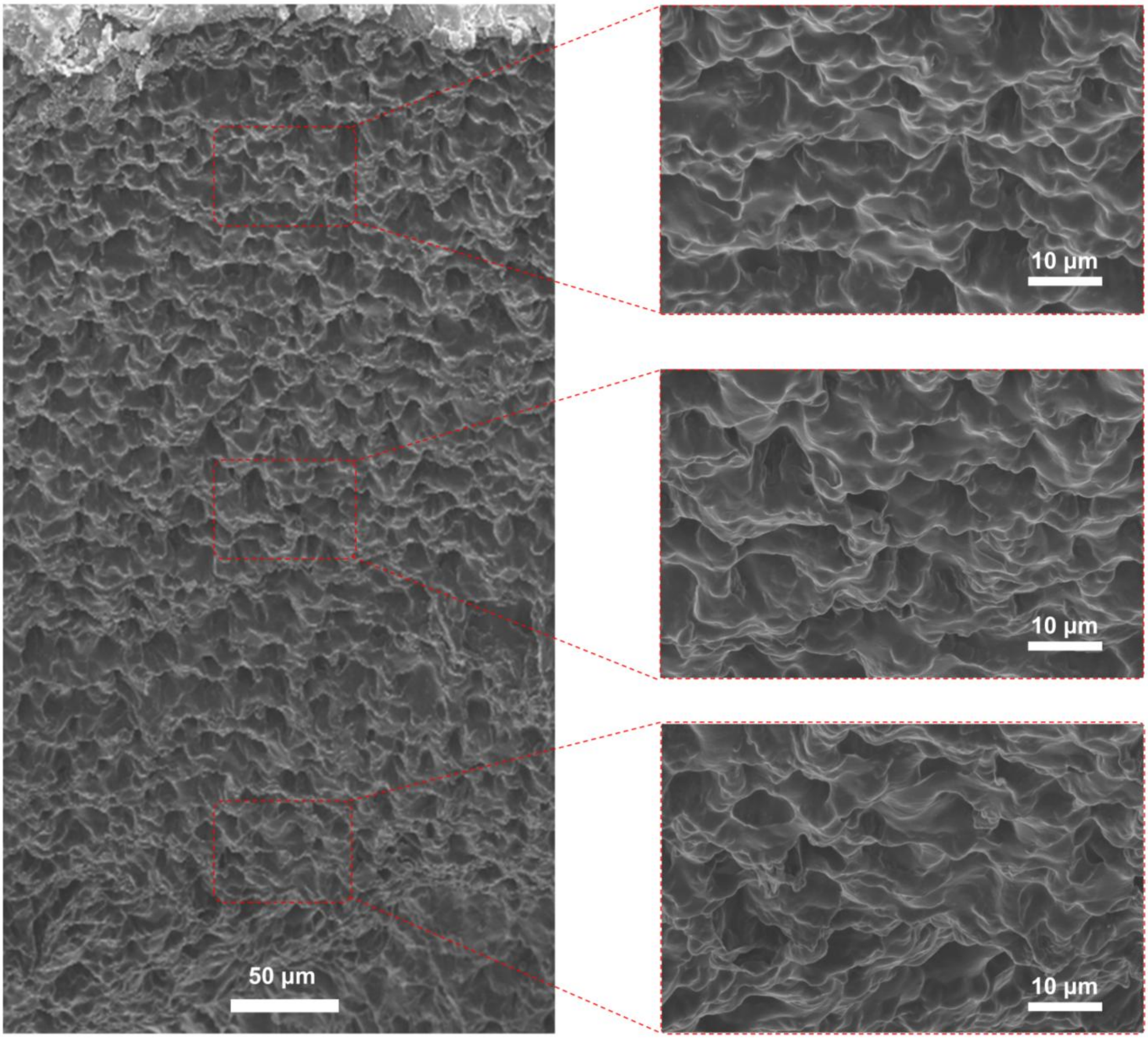

Figure S7. Cross-section SEM images of the NR-PZW hydrogel.

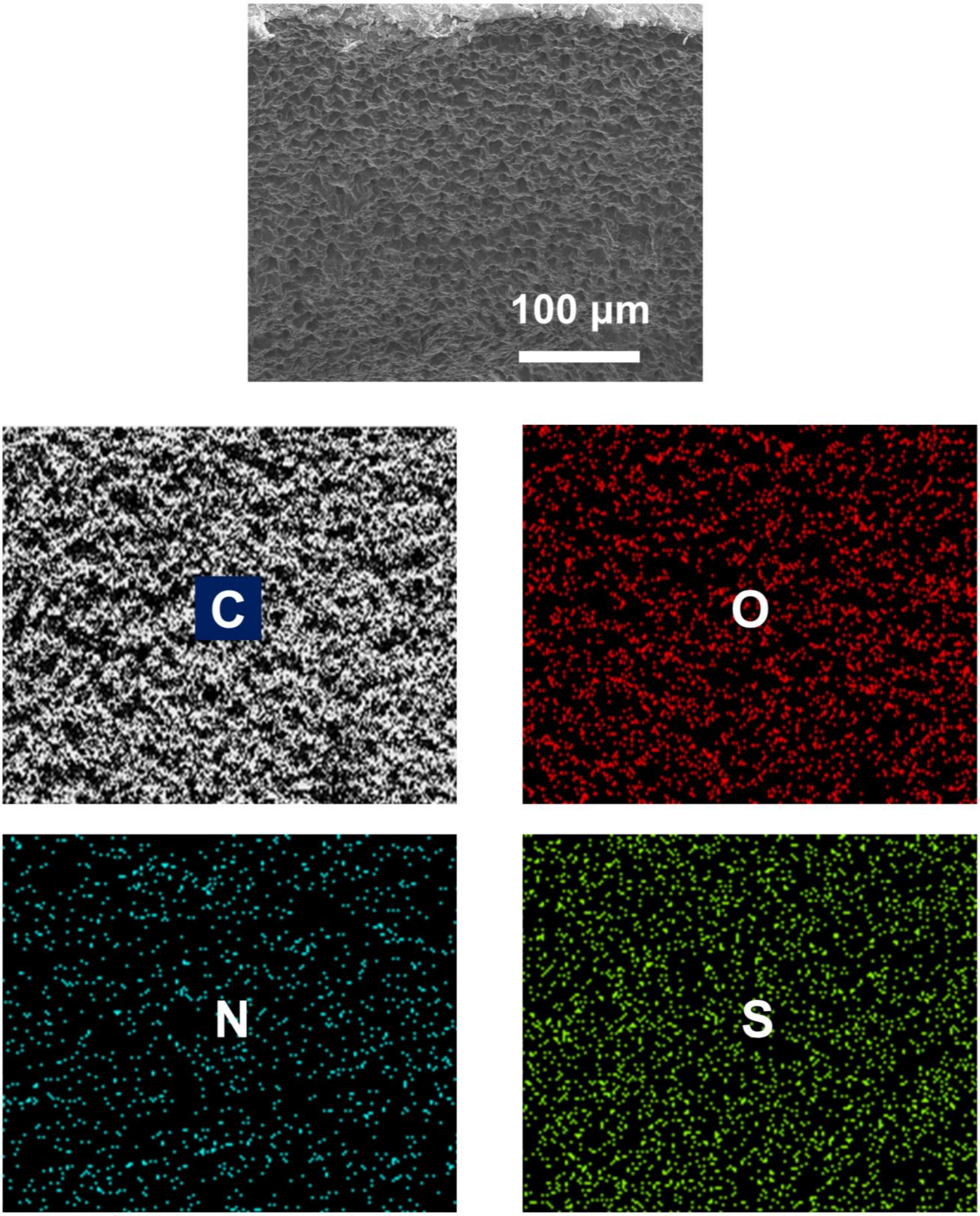

Figure S8. Cross-section SEM images of the NR-PZW hydrogel.

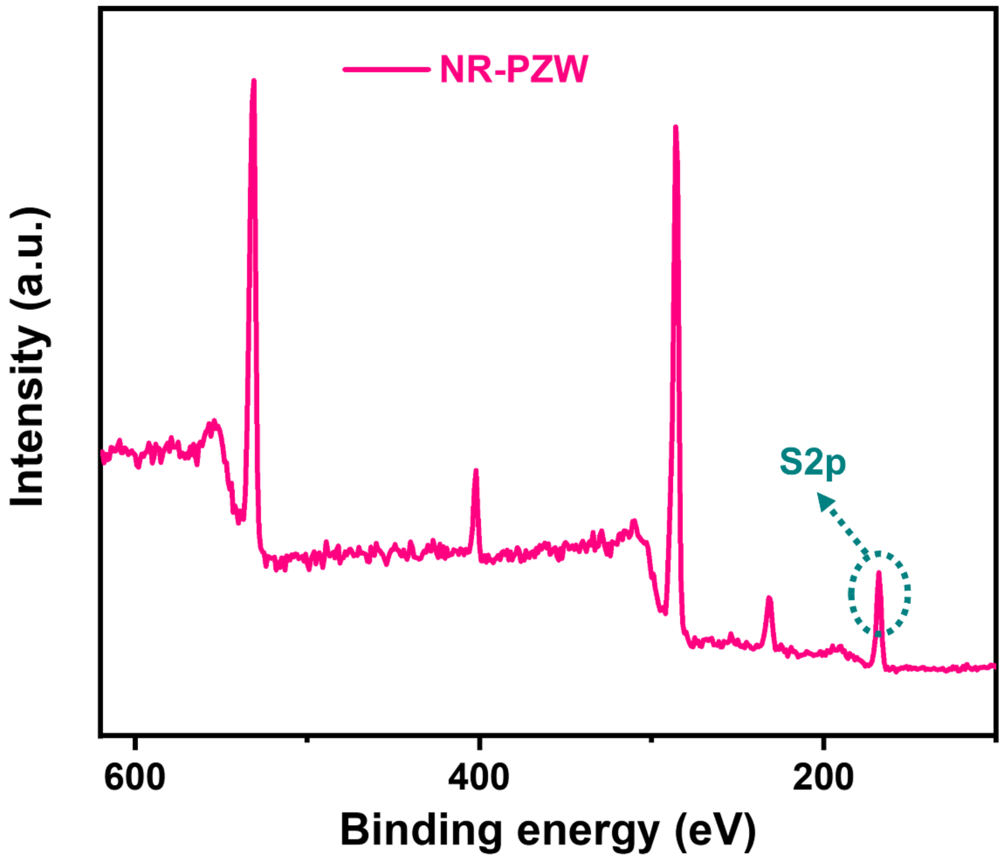

Figure S9. XPS full spectrum of NR-PZW hydrogel.

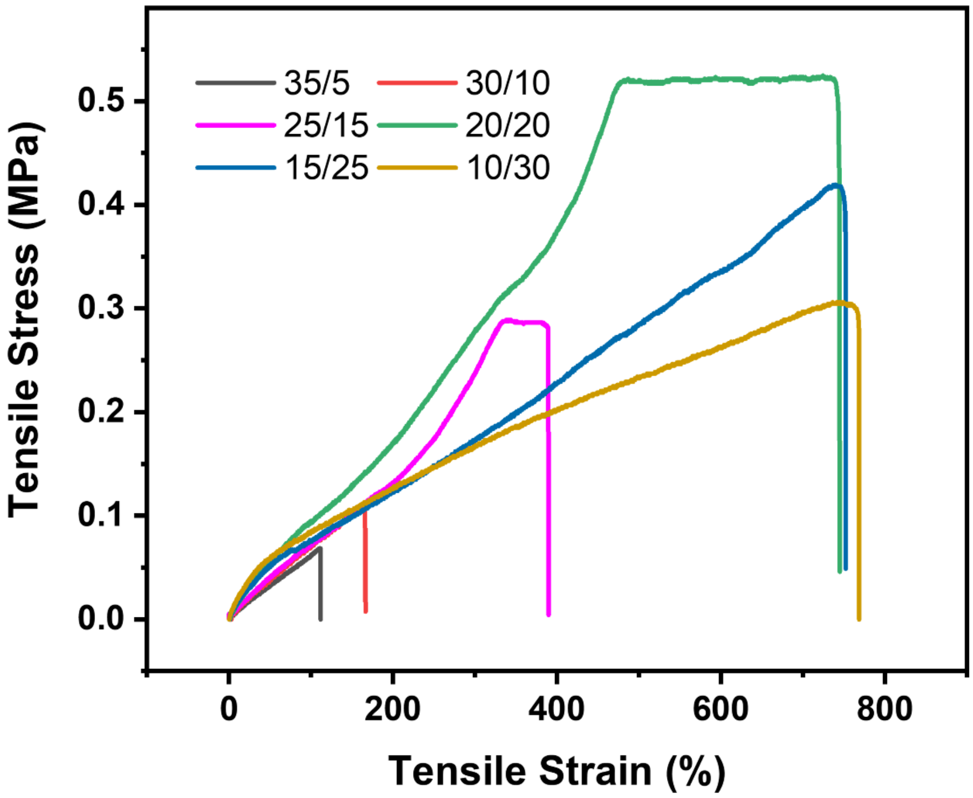

Figure S10. Comparison of tensile properties between the NR-PZW composite hydrogels with different dry mass ratios of the SBMA: natural rubber.

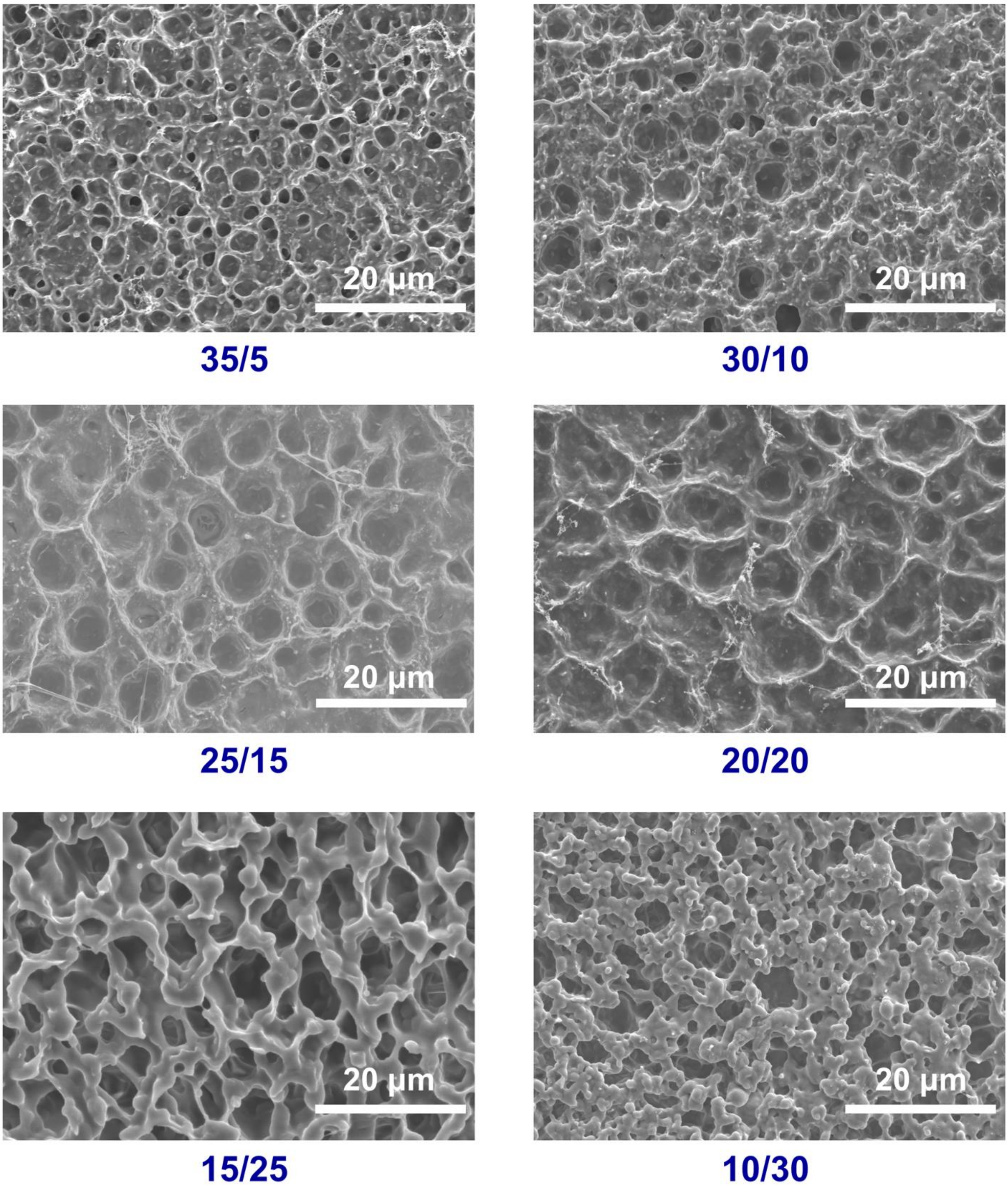

Figure S11. SEM images of PZW, NR, and different NR-PZW.

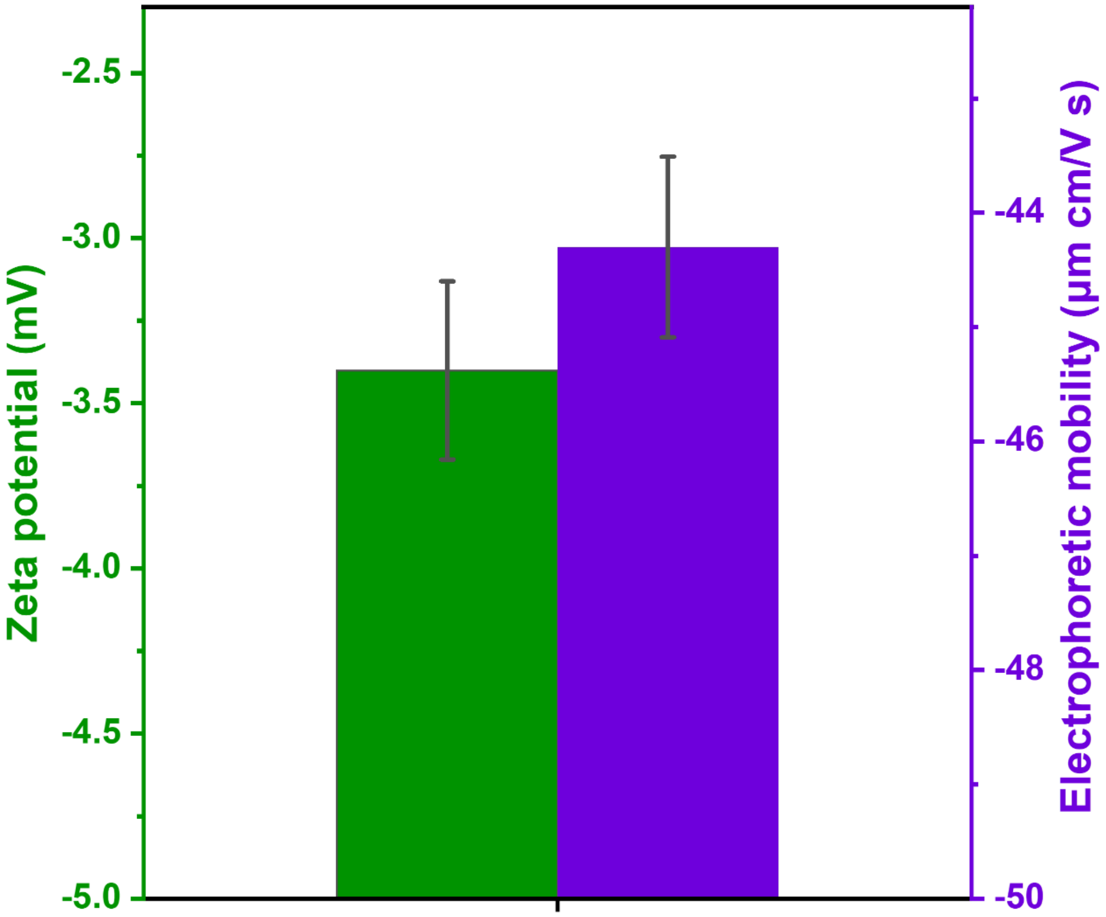

Figure S12. Zeta potential and electrophoretic mobility of the 20 wt% natural rubber aqueous dispersion.

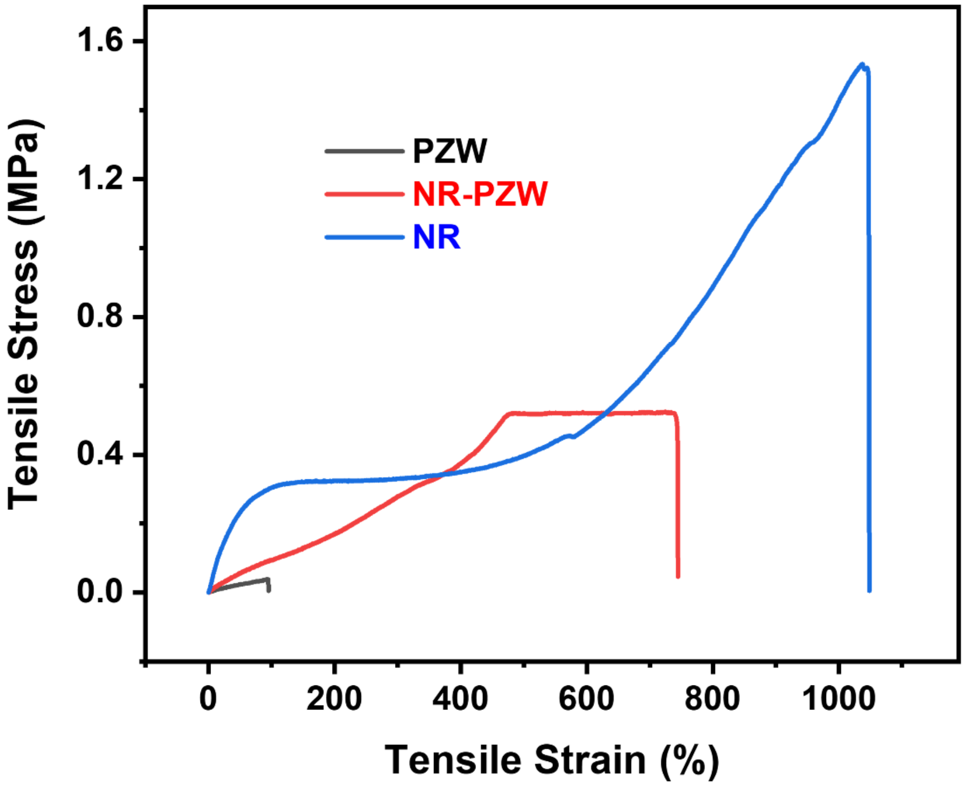

Figure S13. Comparison of tensile properties between PZW, NR-PZW, and NR.

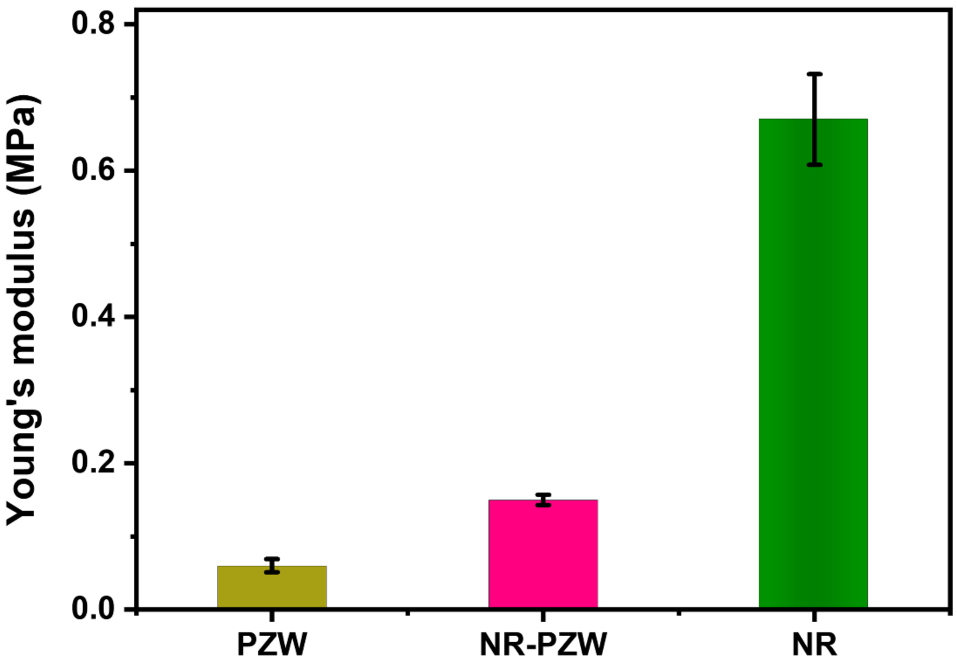

Figure S14. Comparison of Young's modulus between PZW, NR-PZW, and NR.

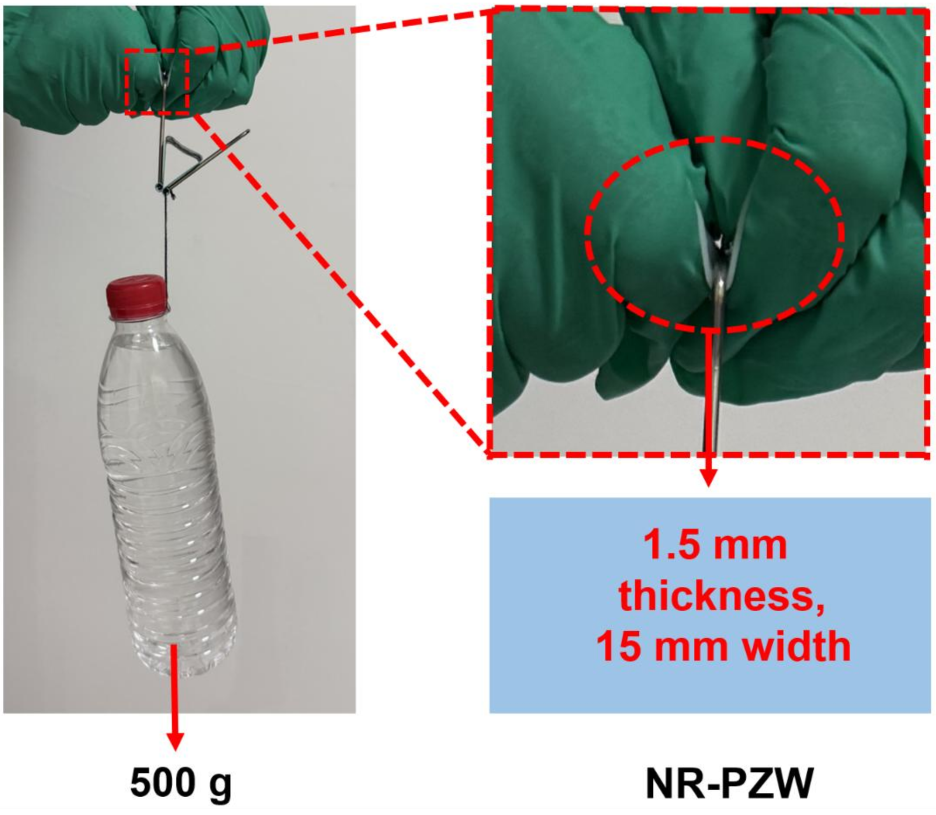

Figure S15. Photos of NR-PZW lifting heavy objects.

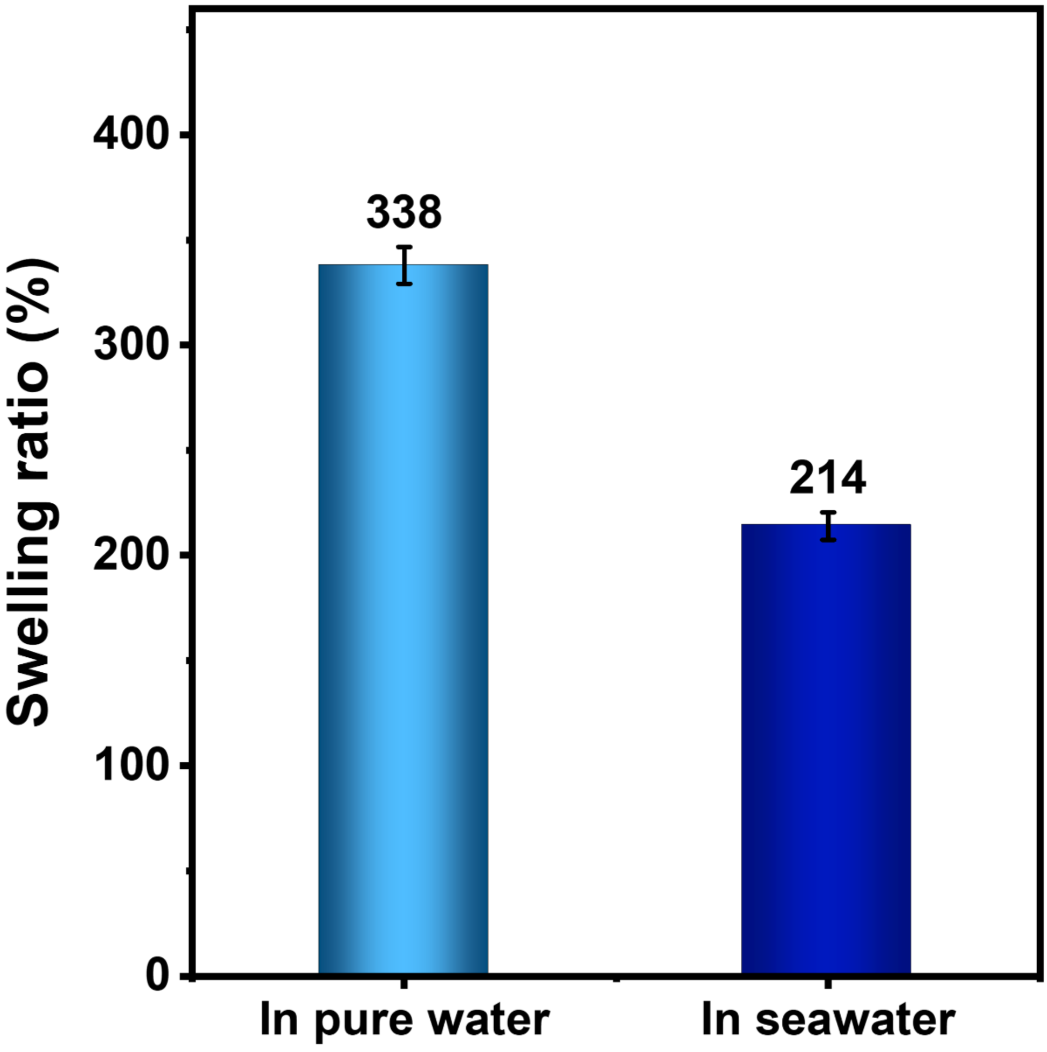

Figure S16. Comparison of swelling ratio of NR-PAM hydrogel between pure water and seawater.

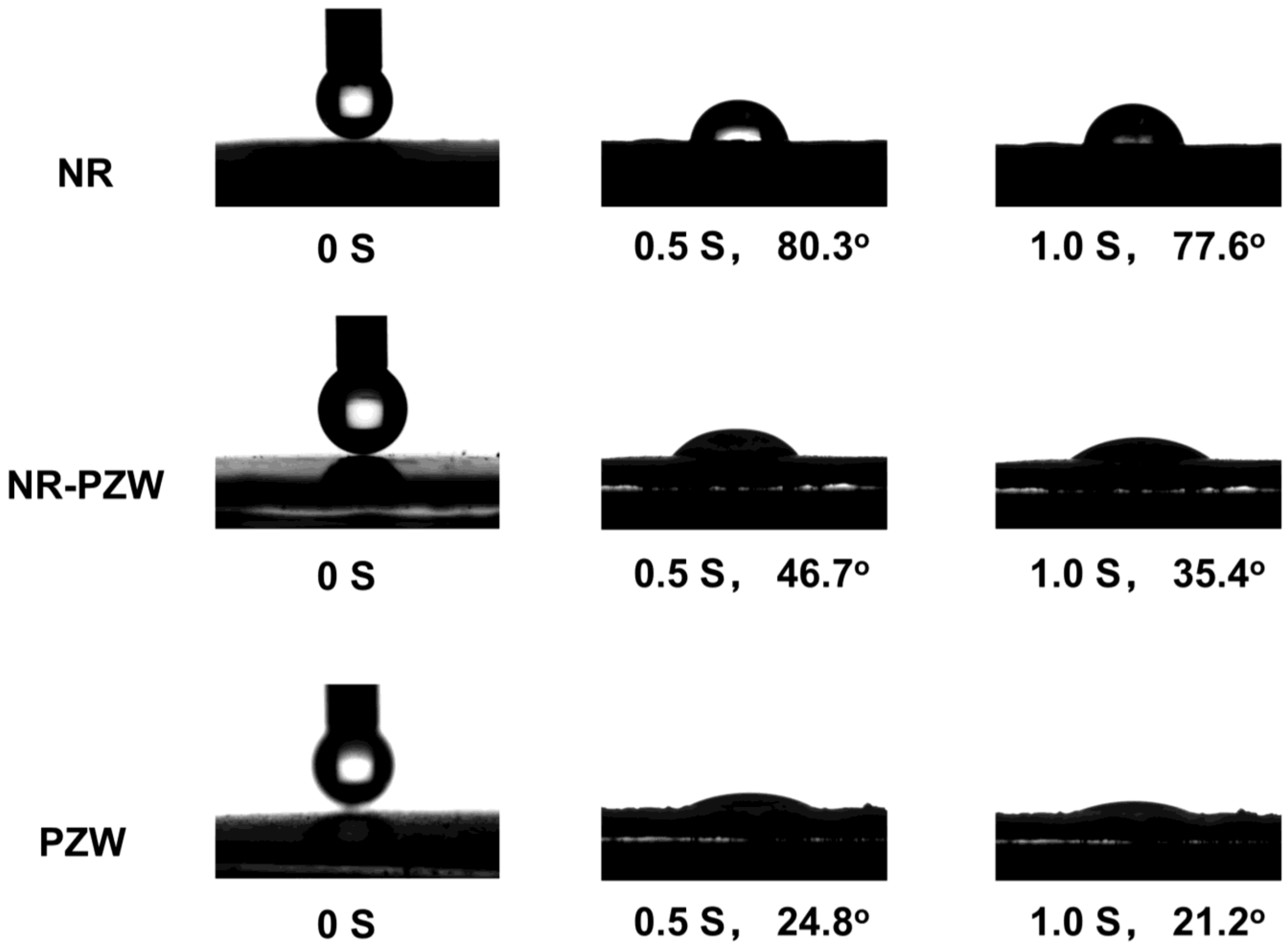

Figure S17. Comparison of seawater contact angle change between the NR, NR-PZW, and PZW samples.

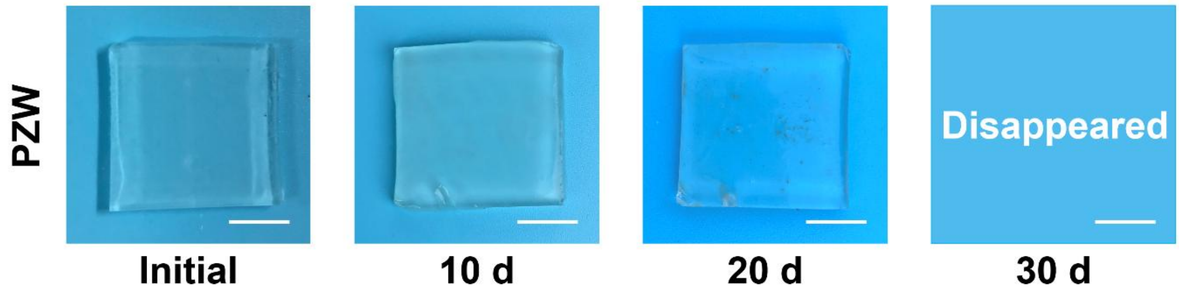

Figure S18. Marine experiment photos of PZW (scale bar: 3.0 cm).

Supporting Tables

Table S1. Formulations of different NR-PZW hydrogels.

sample	SBMA (mg)	NR (mg)	BIS (mg)	APS (mg)
1	35	5	0.35	0.35
2	30	10	0.3	0.3
3	25	15	0.25	0.25
4	20	20	0.2	0.2
5	15	25	0.15	0.15
6	10	30	0.1	0.1

Table S2. Comparison of swelling ratio and water content of NR-PZW hydrogel in pure water and seawater.

Sample	SR	Water content (wt%)	Dry gel content (wt%)
In water	210.0 ± 8.2	52.4 ± 0.4	47.6 ± 0.8
In seawater	605.3 ± 13.2	83.5 ± 1.0	16.5 ± 0.2
